# A Multi-View Heterogeneous Dual-Stream Graph Autoencoder for Predicting circRNA–Disease Associations

**DOI:** 10.3390/biology15212001

**Published:** 2026-11-04

**Authors:** Yu-Hao Zhou, Chang-Qing Yu, Si-Qi Yao, Lei Wang, Zhu-Hong You

**Affiliations:** 1 School of Electronic Information, Xijing University, Xi’an 710123, China; 2 Guangxi Key Lab of Human-Machine Interaction and Intelligent Decision, Guangxi Academy of Sciences, Nanning 530007, China; 3 School of Computer Science and Technology, China University of Mining and Technology, Xuzhou 221116, China; 4 School of Computer Science, Northwestern Polytechnical University, Xi’an 710129, China

**Keywords:** CDA prediction, multi-view graph attention network, heterogeneous relation enhancement, dual-stream graph autoencoder, contrastive learning constraints

## Abstract

Circular RNAs (circRNAs) participate in gene regulation and disease-associated processes. Computational prediction of circRNA–disease associations (CDAs) can help prioritize candidates for biological investigation. Here, we propose a multi-view heterogeneous dual-stream graph autoencoder (MHDA) for CDA prediction. MHDA combines multi-view graph attention encoding, heterogeneous relation enhancement, dual-stream association reconstruction, and contrastive regularization. The encoder learns representations from complementary similarity views and adaptively fuses them. A heterogeneous attention module then integrates within-type and cross-type relationships. The decoder combines element-wise interactions with concatenated pair representations through gated fusion to score candidate associations. On the CircR2Disease dataset, MHDA achieves an AUC of 0.9633 and an AUPR of 0.9599 under five-fold cross-validation. Ablation analyses support the contributions of the main model components. Of the top 25 ranked candidate associations, 22 have supporting evidence in the published literature. These results suggest that MHDA is useful for prioritizing potential CDAs and provides promising candidates for subsequent experimental validation.

## Simple Summary

Circular RNAs are ring-shaped RNA molecules associated with many diseases. Identifying these associations can help researchers select targets for laboratory studies, but testing every possible association is difficult. We developed a computational model that combines similarities between circular RNAs and between diseases with their known associations. The model learns patterns from these connected data and ranks candidates for further investigation. It showed promising benchmark performance, and published studies supported 22 of the 25 highest-ranked candidates examined. A liver cancer example connects the model’s network context with published molecular findings. This approach may help prioritize candidates, while laboratory experiments remain necessary to establish their biological functions.

## 1. Introduction

CircRNAs are endogenous RNA molecules generated through back-splicing and characterized by covalently closed structures [1]. Their circular configuration can confer resistance to exonuclease-mediated degradation [2]. Studies have linked circRNAs to gene regulation and disease-associated processes through interactions with RNAs and proteins [3,4]. For example, circFndc3b interacts with FUS and modulates VEGF-A signaling, reducing cardiomyocyte apoptosis and supporting angiogenesis and cardiac repair after myocardial infarction [5]. In bladder cancer, exosomal circRNA_0013936 enhances the immunosuppressive activity of polymorphonuclear myeloid-derived suppressor cells by regulating FATP2 and RIPK3 expression [6]. These findings support the study of circRNAs as disease-associated regulators and potential biomarkers.

Experimental characterization of RNA function draws on transcriptomic and structural analyses together with targeted molecular assays [7,8]. For circRNAs, such experiments provide essential evidence of expression, molecular interactions, and biological function, but comprehensive testing of candidate disease associations requires substantial time and resources. Computational models can help prioritize candidates by learning patterns from known associations and complementary biological information, thereby guiding subsequent experimental investigation.

Computational approaches to CDA prediction include similarity propagation, matrix-based learning, and graph representation learning. Similarity-based methods infer candidate associations from related circRNAs and diseases, while matrix-based methods recover latent structure from observed association patterns. RNMFLP, for example, combines robust nonnegative matrix factorization with label propagation [9]. Graph-based models offer a framework for integrating heterogeneous biomedical entities and relationships [10]. In CDA prediction, HGECDA integrates circRNA, disease, and miRNA relationships through heterogeneous graph embedding [11], whereas KGETCDA uses a Transformer-based knowledge graph encoder [12]. MNDCDA combines multisource domain-aware embeddings with a feature projection network [13], and KGANCDA learns representations using knowledge graph attention [14]. These approaches illustrate different ways to combine association topology with complementary biological information.

Related circRNA–miRNA interaction studies have explored Transformer–graph attention integration [15], knowledge graph-enhanced language models [16], and multi-hop structural modeling [17]. More recent approaches include variational hypergraph learning with adversarial optimization in VAGAR [18] and Transformer-driven graph fusion [19]. Although these studies address a different interaction target, they provide relevant examples of combining molecular information and graph structure for RNA interaction prediction.

**Figure 1:**
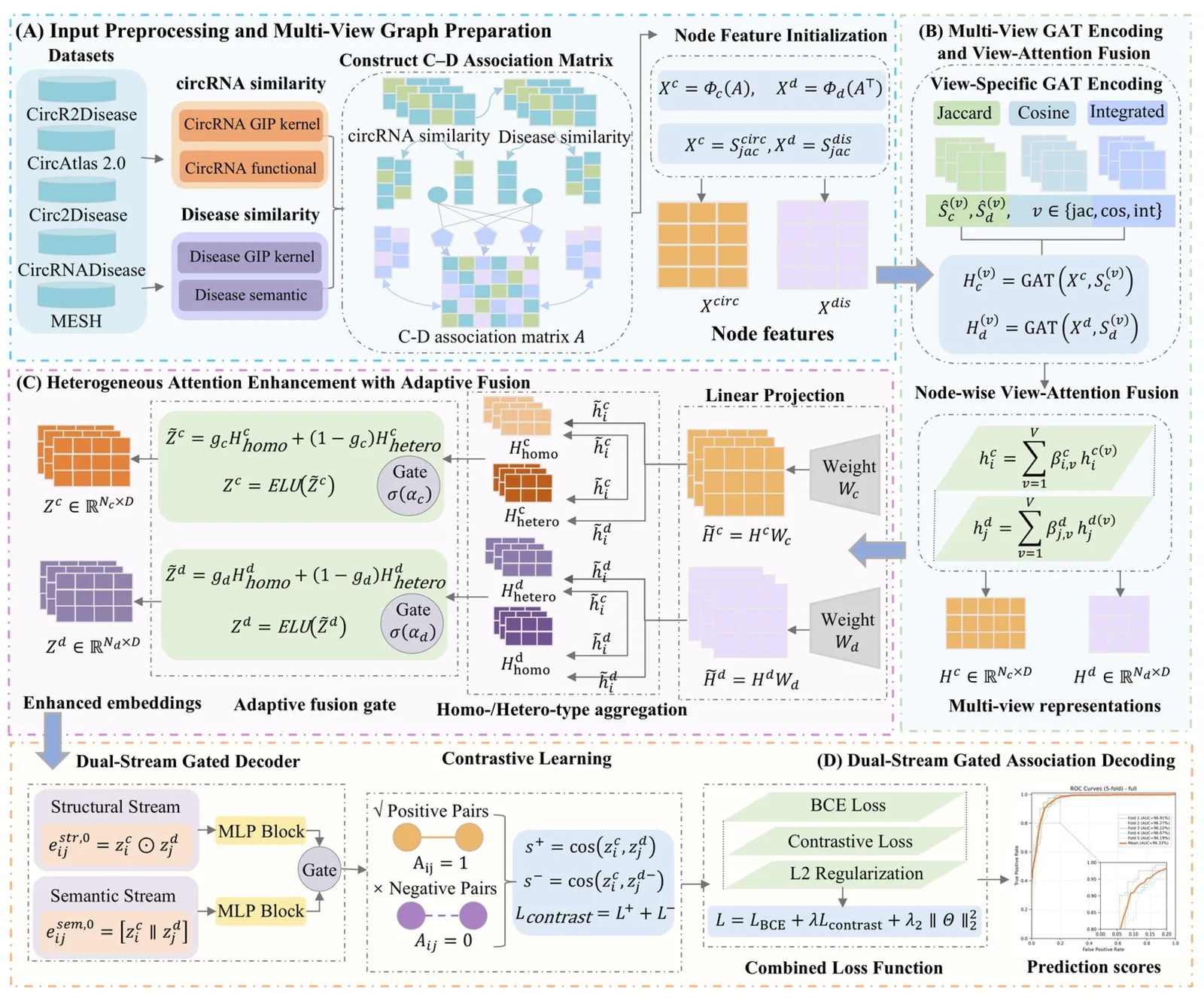
Overall framework of MHDA for CDA prediction. (**A**) Construction of multi-view similarity networks for circRNAs and diseases, accompanied by node feature initialization. (**B**) Parallel representation learning and adaptive fusion of multi-view information through a multi-view graph attention encoding module. (**C**) Joint modeling of intra-type and inter-type relationships via a heterogeneous attention enhancement module. (**D**) Reconstruction of candidate associations from both structural interaction and semantic complementarity perspectives using a dual-stream decoder, integrated with contrastive learning constraints for joint optimization.

The design of MHDA focuses on three aspects of CDA representation learning. First, multiple similarity views can describe complementary relationships between nodes, motivating their joint encoding rather than reliance on a single representation [20]. Second, circRNA–disease networks contain both within-type and cross-type relationships, which motivates coordinated heterogeneous graph learning [21]. Third, a candidate pair can be represented through element-wise interactions and through the joint context retained by concatenation; MHDA uses both operations in a gated decoder. Representation regularization is also included to constrain learning from sparse association data [22]. Accordingly, MHDA combines multi-view graph attention encoding, heterogeneous attention enhancement, dual-stream gated decoding, and a contrastive objective. The encoder learns and fuses view-specific representations, the heterogeneous module integrates the two node types, and the decoder scores candidate associations using complementary pair representations.The framework of MHDA is shown in Figure 1.

The main contributions are threefold. First, MHDA brings multi-view encoding, heterogeneous relation modeling, and association reconstruction into a common framework. Second, its graph attention encoder and heterogeneous enhancement module combine complementary similarity views with within-type and cross-type relationships. Third, a gated dual-stream decoder integrates element-wise and concatenated pair representations, with contrastive regularization included in the training objective.

## 2. Materials and Methods

### 2.1. Dataset

With the continuous accumulation of experimentally validated CDA data, several public databases have provided a crucial foundation for computational prediction studies. In this work, CDA data were collected, curated, and standardized from four publicly available databases: CircR2Disease [23], CircAtlas v2.0 [24], Circ2Disease [25], and CircRNADisease [26], with their statistical details summarized in Table 1. Considering data quality, scale, and representativeness, CircR2Disease—containing both high-throughput sequencing records and experimentally validated associations—was chosen as the primary benchmark dataset for subsequent model construction and performance evaluation [27]. Based on this, a CDA adjacency matrix was constructed to provide a unified data foundation for similarity computation, node representation learning, and model training.

### 2.2. Input Preprocessing and Similarity Construction

Let $A^{\mathrm{full}}\;\in \;R^{n_{c}\;\times \;n_{d}}$ denote the benchmark circRNA–disease association matrix, where *n_c_* and *n_d_* are the numbers of circRNAs and diseases. An entry of 1 indicates a known association, whereas 0 indicates an unobserved pair rather than a confirmed biological negative. Data splitting precedes similarity construction. In each cross-validation fold, a training matrix A is formed using only the positive associations assigned to model training; validation and test associations are excluded. Throughout Section 2.2, Section 2.3 and Section 2.4, A denotes this fold-specific training matrix. All association-derived similarities and node features are constructed from A separately for each fold, while the supplied disease semantic similarity matrix is retained as a fixed input.

#### 2.2.1. Disease Gaussian Interaction Profile Kernel Similarity

Disease GIP similarity quantifies the resemblance between circRNA association profiles. For disease *d_i_*, its interaction profile is the i-th column of the fold-specific training matrix A. Both the interaction profiles and the kernel bandwidth are calculated from this matrix:

(1) *IP*(*d_i_*) = *A*(:, *i*)

Based on the disease interaction profile vectors, the similarity between diseases *d_i_* and *d_j_* is further computed using a Gaussian kernel function:

(2) $$S_{\mathrm{GIP}}^{d}\left(i,j\right)=\exp\left(-\gamma_{d}{∥\mathrm{IP}\left(d_{i}\right)-\mathrm{IP}\left(d_{j}\right)∥}^{2}\right)$$

where ∥⋅∥ denotes the Euclidean norm, and *γ_d_* is the kernel bandwidth parameter, which controls the scale at which similarity decays as the discrepancy between interaction profiles increases. It is defined as

(3) $$\gamma_{d}=\frac{1}{\frac{1}{n_{d}}\sum_{i=1}^{n_{d}}{∥\mathrm{IP}\left(d_{i}\right)∥}^{2}}$$

This results in the disease GIP similarity matrix $S_{\mathrm{GIP}}^{d}$, which primarily captures latent similarities between diseases from the perspective of the underlying association network structure.

#### 2.2.2. circRNA Gaussian Interaction Profile Kernel Similarity

CircRNA GIP similarity is calculated from disease association profiles, reflecting the premise that circRNAs with similar disease associations may have related functions [28]. For circRNA *c_i_*, the interaction profile is the i-th row of the fold-specific training matrix A, namely *IP*(*c_i_*) = *A*(*i*, :). The profiles and kernel bandwidth are recalculated within each fold. The Gaussian kernel similarity between *c_i_* and *c_j_* is defined as

(4) $$S_{\mathrm{GIP}}^{c}\left(i,j\right)=\exp\left(-\gamma_{c}{∥\mathrm{IP}\left(c_{i}\right)-\mathrm{IP}\left(c_{j}\right)∥}^{2}\right)$$

where ∥⋅∥ denotes the Euclidean norm, and *γ_c_* is the kernel bandwidth parameter, which controls the scale at which similarity decays as the discrepancy between interaction profiles increases. It is defined as

(5) $$\gamma_{c}=\frac{1}{\frac{1}{n_{c}}\sum_{i=1}^{n_{c}}{∥\mathrm{IP}\left(c_{i}\right)∥}^{2}}$$

This results in the circRNA GIP similarity matrix $S_{\mathrm{GIP}}^{c}$, which primarily captures latent similarities among circRNAs from the perspective of the underlying association network structure.

#### 2.2.3. Disease Semantic Similarity

Disease semantic similarity is represented using the Medical Subject Headings (MeSH) hierarchy and a directed acyclic graph (*DAG*), as employed in recent circRNA–disease association studies [29]. Each disease d is represented as a *DAG*:

(6) *DAG*(*d*) = (*d*, *T*(*d*), *E*(*d*))

Here, *T(d)* contains disease *d* and its ancestor nodes, and *E(d)* contains the corresponding edges. *C_d_*(*t*) denotes the recursive contribution of node *t* to disease *d* in the first disease-semantic formulation described in [29]. The overall semantic value of disease *d* is the sum of these contributions over its *DAG*:

(7) $$\mathrm{SV}\left(d\right)=\sum_{t\in T\left(d\right)}C_{d}\left(t\right)$$

On this basis, the semantic similarity between diseases *d_i_* and *d_j_* can be computed according to the contributions of the nodes shared by their respective *DAGs*:

(8) $$S_{d}^{\mathrm{Sem}}\left(i,j\right)=\frac{\sum_{t\in T\left(d_{i}\right)\cap T\left(d_{j}\right)}\left(C_{d_{i}}\left(t\right)+C_{d_{j}}\left(t\right)\right)}{\mathrm{SV}\left(d_{i}\right)+\mathrm{SV}\left(d_{j}\right)}$$

where the numerator quantifies the joint contribution of the two diseases from their shared semantic ancestor nodes, while the denominator normalizes the overall semantic information of both diseases. This results in the disease semantic similarity matrix $S_{\mathrm{Sem}}^{d}$, which complements disease similarity information from a semantic perspective.

#### 2.2.4. circRNA Functional Similarity

CircRNA functional similarity assumes that circRNAs associated with semantically related diseases may have similar biological functions [30]. Let *D*(*c_i_*) denote the diseases connected to circRNA *c_i_* by positive entries in the fold-specific training matrix A. Functional similarity is calculated by matching these training-associated disease sets using the fixed disease semantic similarity matrix:

(9) $$S_{c}^{\mathrm{Fun}}\left(i,j\right)=\frac{\sum_{d_{p}\in D\left(c_{i}\right)}\max_{d_{q}\in D\left(c_{j}\right)}S_{d}^{\mathrm{Sem}}\left(d_{p},d_{q}\right)+\sum_{d_{q}\in D\left(c_{j}\right)}\max_{d_{p}\in D\left(c_{i}\right)}S_{d}^{\mathrm{Sem}}\left(d_{q},d_{p}\right)}{\left|D\left(c_{i}\right)\right|+\left|D\left(c_{j}\right)\right|}$$

Here, |*D*(*c_i_*)| *and* |*D*(*c_j_*)| denote the sizes of the two training-associated disease sets. The numerator sums the best semantic matches in both directions, and the denominator normalizes by their combined size. If either disease set is empty, the functional similarity is set to zero. The resulting matrix is denoted by *S^Func^*, or CFS.

#### 2.2.5. Multi-Similarity Integration

GIP similarity reflects association profiles, whereas disease semantic similarity and circRNA functional similarity provide complementary information [31]. C_GIP denotes circRNA GIP similarity calculated from the training association profiles. CFS represents circRNA functional similarity, obtained by comparing the disease sets associated with circRNAs using disease semantic similarity. I_CIRC is the integrated circRNA similarity matrix, which retains positive CFS values and uses C_GIP where CFS is zero. D_GIP denotes disease GIP similarity calculated from the corresponding training association profiles. I_DISE is the integrated disease similarity matrix, which retains positive D_SH values and uses D_GIP for zero entries. D_SH represents disease semantic similarity based on the MeSH hierarchy and the corresponding disease directed acyclic graphs. These representations do not use nucleotide-sequence features. Disease similarity *S_d_* = *I*_*DISE* is constructed by retaining positive semantic similarity values and using D_GIP for zero entries:

(10) $$S_{d}(i,j)\;=\;\left\{\begin{array}{c} D_{\mathrm{SH}}(i,j),\;\;D_{\mathrm{SH}}(i,j)\;>\;0,\\ D_{\mathrm{GIP}}(i,j),\;\;D_{\mathrm{SH}}(i,j)\;=\;0. \end{array}\right.$$

Similarly, circRNA similarity *S_c_* = *I*_*CIRC* retains positive functional similarity values and uses C_GIP for zero entries:

(11) $$S_{c}(i,j)\;=\;\left\{\begin{array}{c} \mathrm{CFS}(i,j),\;\;\mathrm{CFS}(i,j)\;>\;0,\\ C_{\mathrm{GIP}}(i,j),\;\;\mathrm{CFS}(i,j)\;=\;0. \end{array}\right.$$

Thus, integration follows a conditional substitution rule rather than an arithmetic average. The GIP matrices, CFS, and integrated matrices are constructed separately within each fold from the training association matrix and the fixed disease semantic prior. Jaccard and cosine similarities are also calculated from the same training association profiles. Jaccard, cosine, and integrated similarities define the three graph views used by the multi-view encoder.

### 2.3. Multi-View Graph Attention Representation Learning

The multi-view graph attention (GAT) [32] module performs intra-view encoding and inter-view fusion separately for circRNA and disease graphs. Let ${S_{c}}^{v}\;\mathrm{and}\;{S_{d}}^{v}$ denote the similarity matrices for view v ∈ {jac, cos, int}. The initial node feature matrices *X_c_* and *X_d_* consist of the rows of the circRNA and disease Jaccard similarity matrices, respectively. These node features are distinct from the graph views: I_CIRC and I_DISE supply the integrated views, alongside the Jaccard and cosine views. Within each view, GAT aggregates neighborhood information to obtain view-specific representations:

(12) $$H_{c}^{\left(v\right)}=\mathrm{GAT}\left(X^{c},S_{c}^{\left(v\right)}\right),\;H_{d}^{\left(v\right)}=\mathrm{GAT}\left(X^{d},S_{d}^{\left(v\right)}\right)$$

where $H_{c}^{\left(v\right)}\in R^{N_{c}\times D}$ and $H_{d}^{\left(v\right)}\in R^{N_{d}\times D}$ denote the latent representations of circRNA and disease nodes, respectively, under the *v*-th view; *N_c_* and *N_d_* are the numbers of circRNA and disease nodes, respectively, and *D* is the dimensionality of the hidden representation. This process allows the model to simultaneously capture local topological structures and task-relevant representations across different relational views.

Since the importance of different views varies across nodes, simple averaging is insufficient to capture the heterogeneous contributions of individual views. To address this limitation, we introduce a node-level view attention mechanism to adaptively weight and fuse representations derived from multiple views. For circRNA node *c_i_* and disease node *d_j_*, the fused representations are defined as follows:

(13) $$h_{i}^{c}=\sum_{v}\beta_{i,v}^{c}h_{i}^{c\left(v\right)},\;h_{j}^{d}=\sum_{v}\beta_{j,v}^{d}h_{j}^{d\left(v\right)}$$

where $h_{i}^{c(v)}$ and $h_{j}^{d(v)}$ denote the representations of the corresponding nodes under the *v*-th view, while $\beta_{i,v}^{c}$ and $\beta_{j,v}^{d}$ are the normalized view weights, which quantify the extent to which different nodes rely on information from each view. This gives rise to the multi-view fused representation matrices on the circRNA side and the disease side, respectively:

(14) $$H^{c}={\left[h_{1}^{c},h_{2}^{c},\ldots ,h_{N_{c}}^{c}\right]}^{⊤},\;H^{d}={\left[h_{1}^{d},h_{2}^{d},\ldots ,h_{N_{d}}^{d}\right]}^{⊤}$$

Through this design, the model learns node embeddings simultaneously across multiple relational views and adaptively integrates view-specific information according to the characteristics of individual nodes, thereby producing representations that are both more comprehensive and discriminative, subsequently providing high-quality inputs for heterogeneous interaction enhancement.

### 2.4. Heterogeneous Attention Enhancement with Adaptive Fusion

Multi-view encoding captures structural patterns within the circRNA and disease networks. The heterogeneous attention module additionally models circRNA–circRNA, disease–disease, and circRNA–disease relationships in a shared latent space, with an adaptive gate integrating homogeneous and heterogeneous information. The circRNA–disease association block is constructed from the fold-specific training matrix A. Within a fold, training, validation, and testing use the same graph and node features; held-out associations are not added when validation or test pairs are scored. The multi-view representations are projected through type-specific linear transformations:

(15) $${\hat{H}}^{c}=H^{c}W_{c},\;{\hat{H}}^{d}=H^{d}W_{d}$$

where *W_c_* ∈ ℝ*^D^*^×^*^D^* and *W_d_* ∈ ℝ*^D^*^×^*^D^* are learnable projection matrices. Through this mapping, circRNA and disease nodes are projected into a unified latent space of identical dimensionality, providing the foundation for subsequent joint modeling of homogeneous and heterogeneous relationships.

The model aggregates information separately from same-type and cross-type neighborhoods. For circRNA nodes, this produces a homogeneous representation $H_{\mathrm{homo}}^{c}$ and a heterogeneous representation $H_{\mathrm{hetero}}^{c}$; for disease nodes, it produces a homogeneous representation $H_{\mathrm{homo}}^{d}$ and a heterogeneous representation $H_{\mathrm{hetero}}^{d}$. Homogeneous aggregation reinforces local consistency among nodes within the same-type graph, while heterogeneous aggregation explicitly introduces complementary semantic information conveyed through cross-type association propagation. To mitigate the rigidity associated with fixed-proportion fusion, learnable gating parameters are introduced to dynamically balance the two types of information:

(16) $${\tilde{Z}}^{c}=g_{c}H_{\mathrm{homo}}^{c}+\left(1-g_{c}\right)H_{\mathrm{hetero}}^{c}$$

(17) $${\tilde{Z}}^{d}=g_{d}H_{\mathrm{homo}}^{d}+\left(1-g_{d}\right)H_{\mathrm{hetero}}^{d}$$

where *g_c_* and *g_d_* are learnable gating coefficients constrained to the interval [0, 1], which respectively control the extent to which circRNA and disease nodes rely on homogeneous and heterogeneous information. Finally, the enhanced node representations are computed through a nonlinear activation function:

(18) $$Z^{c}=\mathrm{ELU}\left({\tilde{Z}}^{c}\right),\;Z^{d}=\mathrm{ELU}\left({\tilde{Z}}^{d}\right)$$

This module allows the model to dynamically balance same-type structural constraints and cross-type interaction-based complementarity, thereby producing more discriminative representations for downstream CDA reconstruction.

### 2.5. Dual-Stream Gated Association Decoding

To further characterize potential CDAs at the candidate-pair level, we design a dual-stream gated decoding module that jointly models node-pair relationships from structural interaction and semantic complementarity perspectives. Compared with decoding schemes relying on a single pathway for association scoring, this module simultaneously preserves the matching relationship between circRNAs and diseases in the shared latent space, as well as complementary contextual information contained in the representations of both node types, thereby enhancing the expressive power of candidate association representations. For an arbitrary candidate circRNA–disease node pair, let $z_{i}^{c}$ and $z_{j}^{d}$ denote their enhanced representations, respectively. Specifically, the structural stream constructs the pairwise representation via the Hadamard product to capture correspondence between the two node types in the latent space, while the semantic stream employs concatenation to preserve joint contextual information of both nodes, namely:

(19) $$e_{\mathrm{ij}}^{\mathrm{str}}=z_{i}^{c}⊙z_{j}^{d},\;e_{\mathrm{ij}}^{\mathrm{sem}}=\left[z_{i}^{c}∥z_{j}^{d}\right]$$

Subsequently, the structural and semantic streams are each passed through independent nonlinear mappings, yielding the within-stream representations $h_{\mathrm{ij}}^{\mathrm{str}}$ and $h_{\mathrm{ij}}^{\mathrm{sem}}$, respectively. Since candidate node pairs may rely on these two types of information to varying degrees, a pair-level gating mechanism is introduced to adaptively regulate the contribution of the two pathways:

(20) $$g_{\mathrm{ij}}=\sigma \left(W_{g}\left[\;h_{\mathrm{ij}}^{\mathrm{str}}∥h_{\mathrm{ij}}^{\mathrm{sem}}\;\right]+b_{g}\right)$$

(21) $$h_{\mathrm{ij}}^{\mathrm{fuse}}=g_{\mathrm{ij}}⊙h_{\mathrm{ij}}^{\mathrm{str}}+\left(1-g_{\mathrm{ij}}\right)⊙h_{\mathrm{ij}}^{\mathrm{sem}}\;$$

where *g_ij_* denotes the gating vector associated with the candidate node pair. This mechanism allows the model to dynamically balance the relative contributions of structural matching information and semantic complementary information for candidate node pairs, instead of relying on a fixed fusion strategy, thereby enhancing the flexibility and adaptability of the decoding process. Finally, the model computes the association score based on the fused pairwise representation:

(22) $${\hat{A}}_{\mathrm{ij}}=\sigma \left(w_{o}^{⊤}h_{\mathrm{ij}}^{\mathrm{fuse}}+b_{o}\right)\;$$

where ${\hat{A}}_{\mathrm{ij}}$ denotes the predicted probability of association between circRNA *c_i_* and disease *d_j_*, and the predictions for all candidate node pairs collectively form the predicted association matrix $\hat{A}$. This “fusion-before-prediction” design prevents dilution of dual-stream information that may result from simple concatenation at the output stage, thereby allowing the complementary strengths of the structural and semantic streams to be fully exploited, enabling more refined modeling and more accurate prediction of potential CDAs.

### 2.6. Implementation Details and Model Training

MHDA is optimized using Adam with an initial learning rate of 2.8 × 10^−4^, multiplied by 0.95 every 15 epochs. Training runs for 130 epochs with a batch size of 512, a hidden dimension of 28, and a dropout rate of 0.42. Validation is performed every 10 epochs, and the checkpoint with the highest validation AUC is selected. Early-stopping patience is set to 15 consecutive validation evaluations without improvement; with validation every 10 epochs, this condition does not trigger before the 130-epoch limit. The random seed for data splitting and negative sampling is 1024, and the model-training seeds for the five folds are 42–46.

The reconstruction loss ℒ*_rec_* is the mean binary cross-entropy with a label-smoothing coefficient of 0.15 and smoothed targets ỹ = 0.85y + 0.075. The contrastive loss is defined as

(23) $$L_{\mathrm{CL}}=\frac{1}{\left|B^{+}\right|}\sum_{(i,j)\;\in \;B^{+}}{\left(1-s_{\mathrm{ij}}\right)}^{2}+\frac{1}{\left|N^{-}\right|}\sum_{(i,j)\;\in \;N^{-}}{\left[\max(0,s_{\mathrm{ij}})\right]}^{2}$$

Here, *s_ij_* is the cosine similarity between the circRNA and disease embeddings, ℬ^+^ contains the positive training associations in the current minibatch, and 𝒩^−^ contains putative negatives sampled from the training negative pool of the same fold. An empty-set term is assigned a value of zero. The overall objective is

(24) $$L=L_{\mathrm{rec}}+0.018L_{\mathrm{CL}}+\frac{1.5\times 10^{-4}}{2}\sum_{\theta \;\in \;\Theta_{\mathrm{reg}}}{{\left\|\theta \right\|}_{2}}^{2}$$

Here, *Θ_reg_* denotes the parameters subject to L2 regularization. The reconstruction and contrastive terms have weights of 1 and 0.018, respectively. The L2 regularization coefficient is 1.5 × 10^−4^, applied to one-half of the squared parameter norm.

## 3. Results

### 3.1. Evaluation Protocol and Metrics

We use five-fold cross-validation on CircR2Disease. The dataset contains 561 circRNAs and 100 diseases, giving 56,100 possible pairs: 607 known associations and 55,493 unobserved pairs. Known positive associations are partitioned into five folds, with one fold held out for testing in each round. In each fold, 607 unobserved pairs are randomly sampled without replacement and assigned negative labels, corresponding to approximately 1.09% of the unobserved pool. The outer training pool and test set each use a positive-to-negative ratio of 1:1. A stratified 20% subset of the outer training pool is reserved for validation, preserving approximately balanced class proportions. Training, validation, and test pairs are mutually exclusive within each fold. Negative sampling is performed separately for each fold with a fixed random seed.

Only the positive associations assigned to model training are used to construct the input similarities and heterogeneous graph, as described in Section 2.2, Section 2.3 and Section 2.4. The nonzero entries in the training association matrix correspond exactly to the positive associations used for model training. Entries corresponding to validation and test positive associations are zero, so the association matrix used to construct the model inputs excludes the held-out associations.

Performance is assessed using AUC, AUPR, F1, precision, recall, and accuracy (ACC). AUC and AUPR evaluate ranking performance across decision thresholds. The classification threshold is selected by maximizing validation F1 and is then applied to the held-out test set. Comparing the predicted classes with the labels of the evaluated pairs yields the numbers of true positives (TP), false positives (FP), true negatives (TN), and false negatives (FN). Precision, recall, F1, and ACC are defined as follows:

(25) $$\mathrm{Precision}=\frac{\mathrm{TP}}{\mathrm{TP}+\mathrm{FP}}$$

(26) $$\mathrm{Recall}=\frac{\mathrm{TP}}{\mathrm{TP}+\mathrm{FN}}$$

(27) $$F1=2\times \frac{\mathrm{Precision}\times \mathrm{Recall}}{\mathrm{Precision}+\mathrm{Recall}}$$

(28) $$\mathrm{ACC}=\frac{\mathrm{TP}+\mathrm{TN}}{\mathrm{TP}+\mathrm{TN}+\mathrm{FP}+\mathrm{FN}}$$

Results are reported as the mean ± standard deviation across the five folds. AUPR, F1, precision, and recall are interpreted under the specified 1:1 positive-to-negative sampling scheme.

### 3.2. Model Performance Evaluation

**Figure 2:**
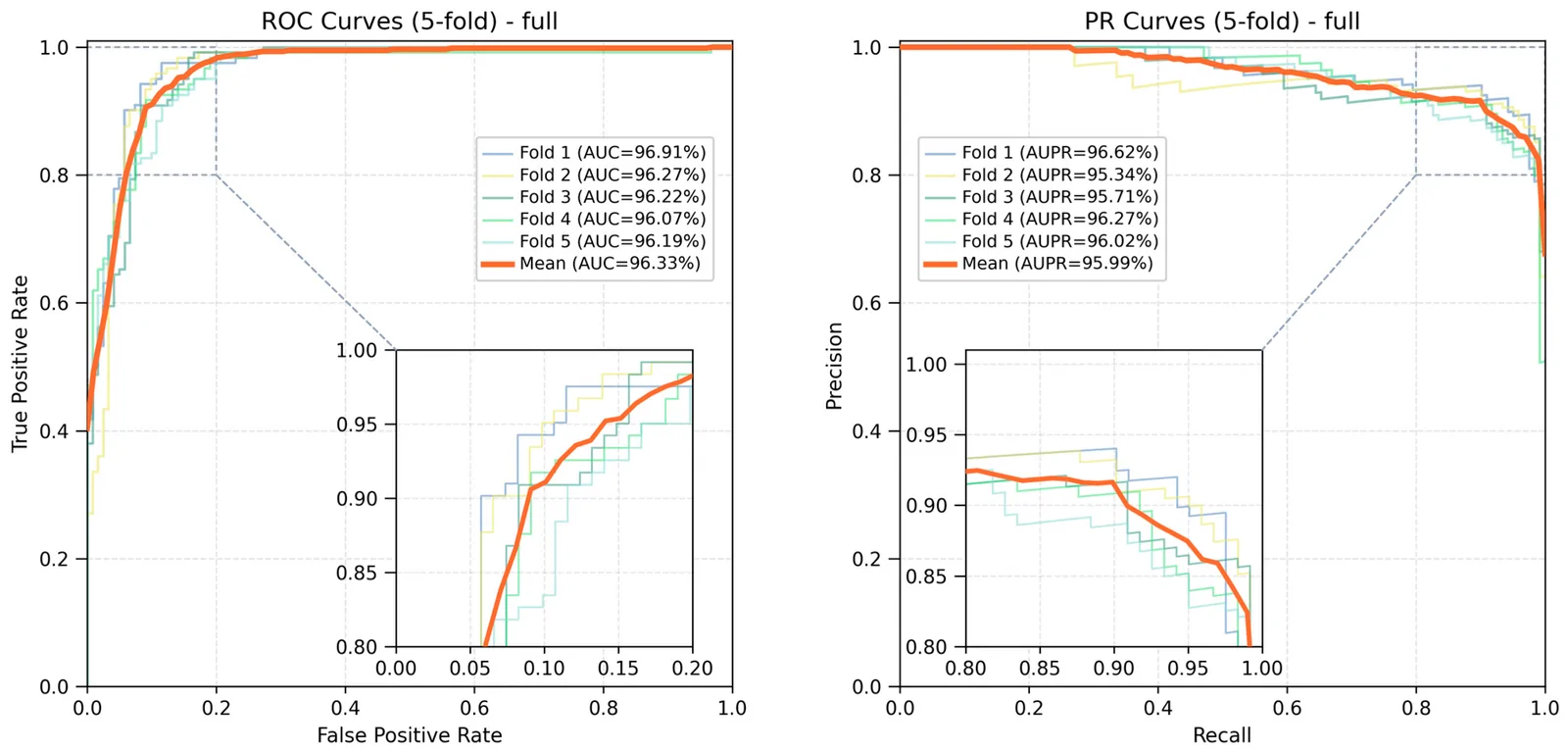
ROC and PR Curves.

MHDA was evaluated on CircR2Disease using five-fold cross-validation. Table 2 reports the results, and Figure 2 shows the ROC and PR curves. The mean AUC and AUPR were 0.9633 and 0.9599, respectively; the corresponding F1, precision, recall, and ACC values were 0.9179, 0.8764, 0.9654, and 0.9134. These results indicate consistent predictive performance across the evaluated folds under the specified sampling and evaluation procedure.

### 3.3. Comparison of Input Preprocessing Feature Combinations

**Figure 3:**
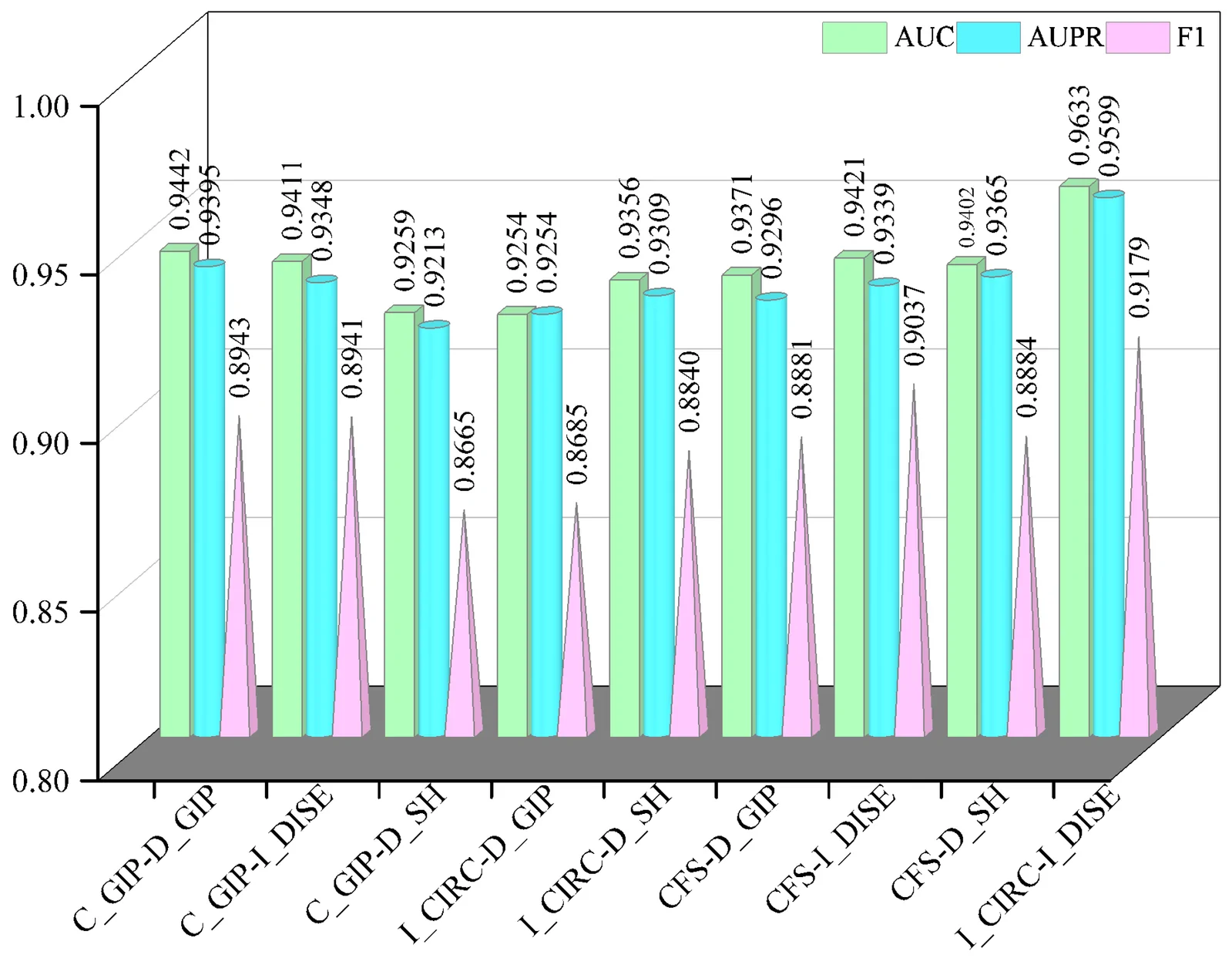
Performance of nine input similarity combinations in terms of AUC, AUPR, and F1. C_GIP: circRNA Gaussian interaction profile similarity; CFS: circRNA functional similarity; I_CIRC: integrated circRNA similarity; D_GIP: disease Gaussian interaction profile similarity; I_DISE: integrated disease similarity; D_SH: disease semantic similarity.

To select the input similarity configuration, we compared nine pairings of circRNA-side and disease-side similarities while keeping the model architecture and training parameters fixed. The circRNA-side options were GIP similarity (C_GIP), functional similarity (CFS), and integrated similarity (I_CIRC); the disease-side options were GIP similarity (D_GIP), semantic similarity (D_SH), and integrated similarity (I_DISE). None of these inputs is a nucleotide-sequence feature. Figure 3 compares the nine pairings in terms of AUC, AUPR, and F1. I_CIRC–I_DISE achieved the best performance among the evaluated combinations and was therefore selected as the default similarity pairing for all subsequent experiments.

### 3.4. Multi-View Ablation Study

**Figure 4:**
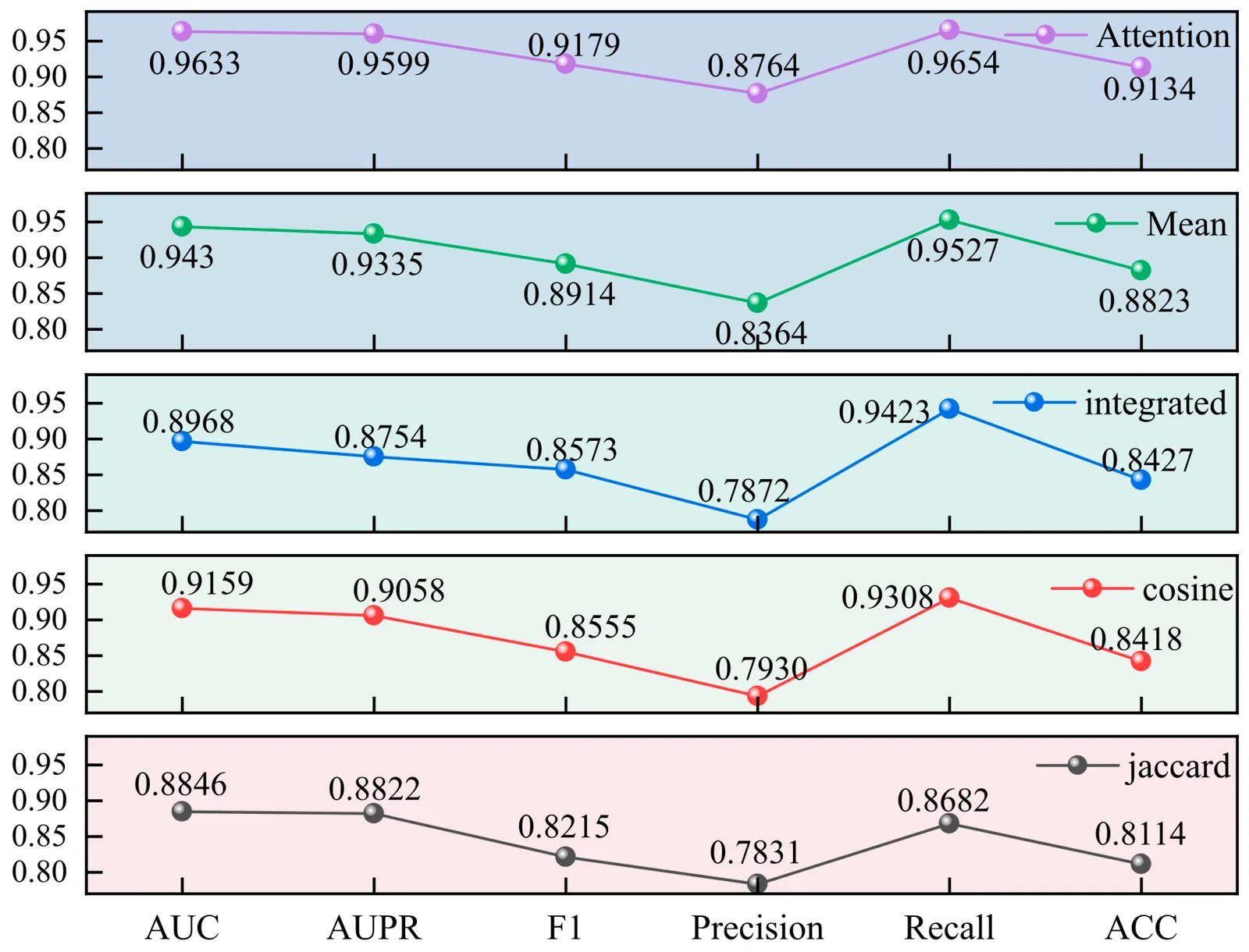
Performance Comparison of the Model under Different Single-View and Multi-View Fusion Strategies.

To further assess the effectiveness of the multi-view encoding module, we compared single-view representation learning with multi-view fusion strategies. Specifically, the single-view settings included three relational views—Jaccard, Cosine, and Integrated—while the multi-view settings employed either simple mean fusion (Mean) or adaptive attention-based fusion (Attention). Figure 4 illustrates the overall performance under these settings. The results indicate that all three single-view models performed worse than the multi-view schemes, suggesting that reliance on a single relational view is insufficient to fully characterize complex CDA, while the joint use of multiple views substantially improves the completeness of learned representations. Furthermore, Attention consistently outperformed Mean across all evaluation metrics, indicating that the contributions of different views to association prediction are heterogeneous. The attention-based adaptive weighting mechanism therefore more effectively exploits complementary information across views, enhancing multi-view integration. Taken together, these findings demonstrate that multi-view encoding significantly contributes to the model’s performance gains and that its adaptive fusion mechanism provides a clear representational advantage over equal-weight fusion.

### 3.5. Ablation Study on the Heterogeneous Layer

**Figure 5:**
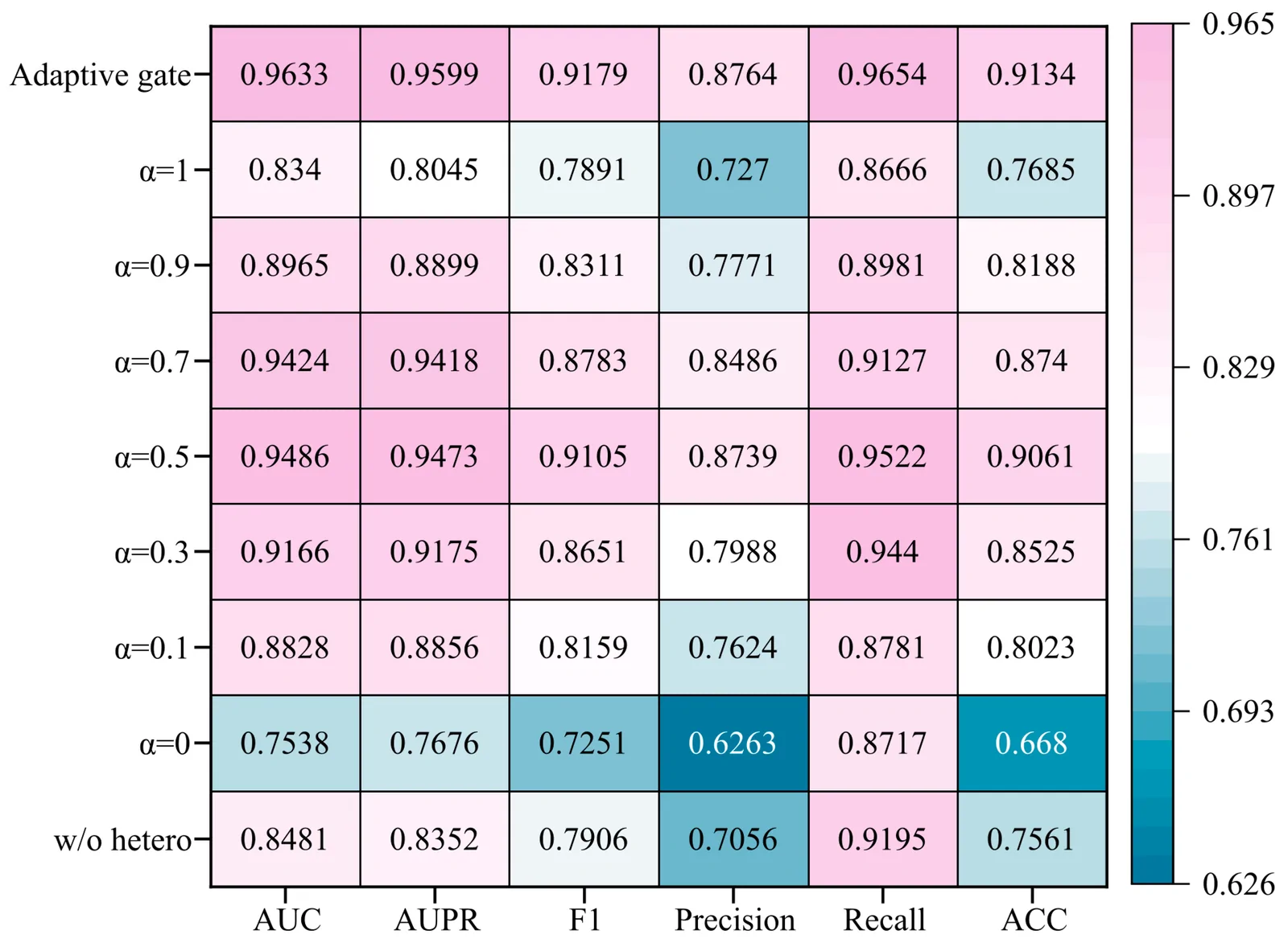
Performance Comparison of the Model under Different Heterogeneous Enhancement Settings and Gating Fusion Strategies.

To assess the practical contribution of the heterogeneous attention enhancement module to association modeling, we compared model performance under different heterogeneous enhancement settings within the multi-view representation learning framework. Specifically, the experimental settings included: a model without the heterogeneous enhancement module (w/o hetero), a model incorporating the heterogeneous layer with a fixed gating coefficient *α*, and a model employing an adaptive gating mechanism (Adaptive Gate). Figure 5 illustrates the comparative results of these settings across evaluation metrics. Overall, the model w/o hetero exhibited inferior performance, indicating that information propagation confined to homogeneous graphs is insufficient to fully exploit cross-type interaction information between circRNAs and diseases. After introducing heterogeneous enhancement, model performance consistently improved. However, results with a fixed *α* varied substantially depending on the manually specified weight, suggesting that the relative importance of homogeneous and heterogeneous information is not constant. By contrast, Adaptive Gate achieved the best results across all evaluation metrics, with AUC, AUPR, and F1 reaching 0.9633, 0.9599, and 91.79%, respectively. This demonstrates that the adaptive gating mechanism more effectively balances the contributions of homogeneous and heterogeneous information.

Furthermore, when *α* takes extreme values, the model degenerates into one that relies exclusively on either homogeneous relations or heterogeneous interactions, and neither scenario achieves optimal performance. This finding suggests that homogeneous relations and cross-type interactions are highly complementary in CDA modeling. Taken together, these results indicate that the heterogeneous enhancement module further improves node representation quality beyond multi-view representation learning alone, and that the adaptive gating mechanism is the key factor enabling this improvement.

### 3.6. Analysis of the Dual-Stream Decoder and Its Internal Classifier

**Figure 6:**
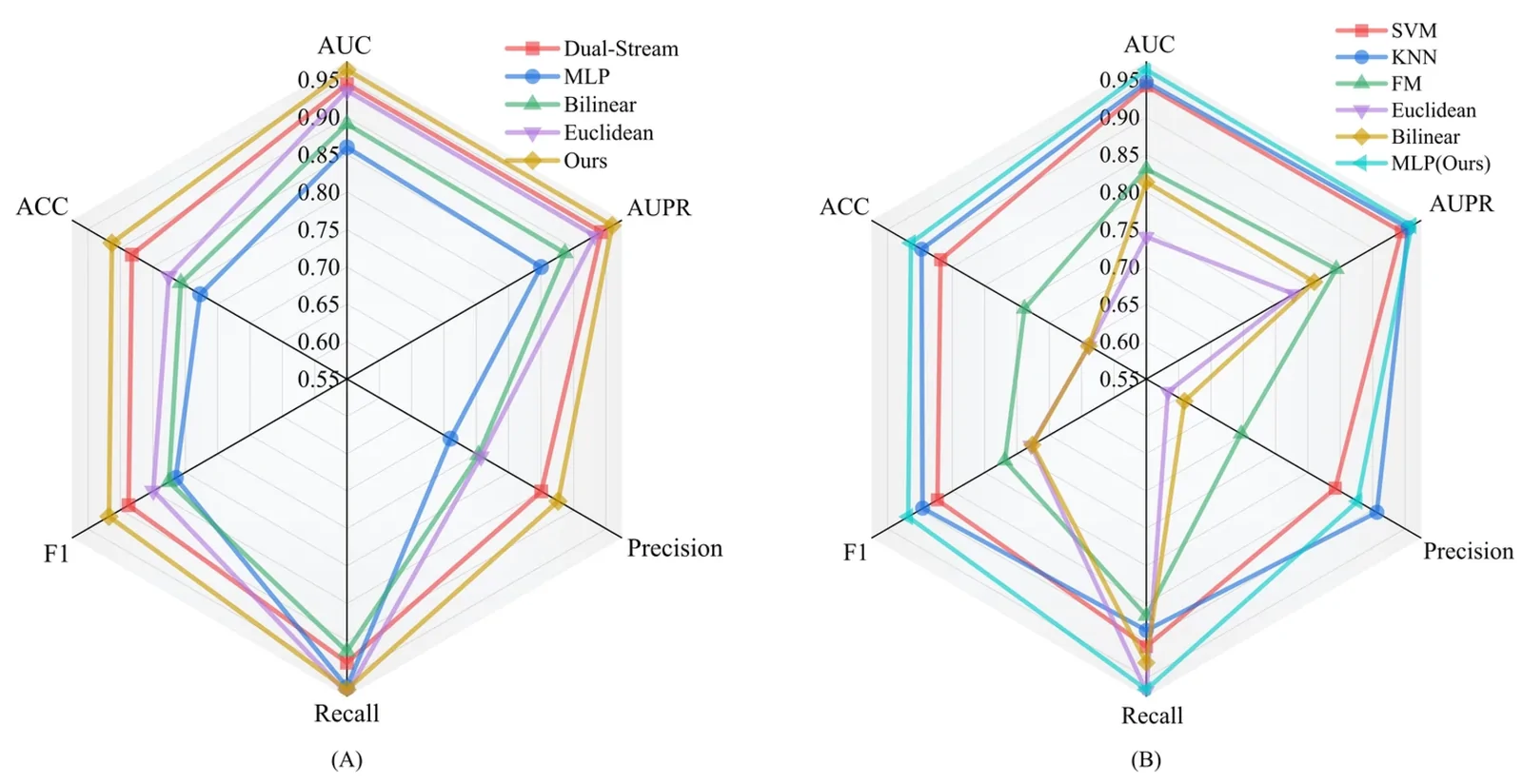
Performance Performance Comparison of the Model under Different Decoder Architectures and In-ternal Classifier Settings. (A) Comparison of different decoder architectures; (B) comparison of different internal classifier settings.

To examine the contribution of decoder design, we compared the original Dual-Stream, MLP, Bilinear, Euclidean, and optimized dual-stream decoders. As shown in Figure 6A, the optimized dual-stream decoder achieved the best overall performance among the evaluated settings. Its element-wise and concatenation streams retain complementary pair information, while the within-stream mappings and gated fusion jointly contribute to association scoring.

We also compared SVM, KNN, FM, Euclidean, Bilinear, and the optimized MLP within the dual-stream framework. Figure 6B shows that the optimized MLP achieved the best overall performance among these classifier settings. Together, these comparisons support the selected combination of the optimized dual-stream decoder and MLP classifier for MHDA.

### 3.7. Comparison with Published Results

Table 3 presents the results obtained for MHDA alongside those reported for seven existing CDA prediction methods on the CircR2Disease benchmark: TCGIBMT [33], Bi-SGTAR [34], HGECDA [11], HGCNCDA [35], GATCL2CD [36], RPMVCDA [37], and MNDCDA [13]. These results provide a reference for the performance reported across the included studies.

### 3.8. Case Study

During model training, the known circRNA–disease associations assigned to the training partition serve as positive samples for learning association patterns. For case-study selection, eligibility is defined against the complete benchmark association matrix: recorded positive associations are excluded, and only unobserved pairs corresponding to zero entries are eligible for prediction and ranking. This criterion covers known positive associations in all cross-validation partitions. The case-study candidate pool is distinct from the held-out test set used in cross-validation. Eligible pairs are scored and ranked for literature assessment. Table 4 summarizes literature support for 25 ranked candidates: 22 had supporting evidence in published studies, whereas three lacked identified literature support at the time of retrieval. These findings provide literature-based context for candidate prioritization; they do not constitute direct experimental validation of the predictions.

Representative literature-supported candidates include hsa_circ_0001727 (circZKSCAN1) in hepatocellular carcinoma [38] and circ-FBXW7 in lung adenocarcinoma [39]. The corresponding studies link these circRNAs to tumor-related biological processes, providing context for the biological plausibility of the ranked associations. Candidates without identified literature support remain hypotheses for further investigation.

**Figure 7:**
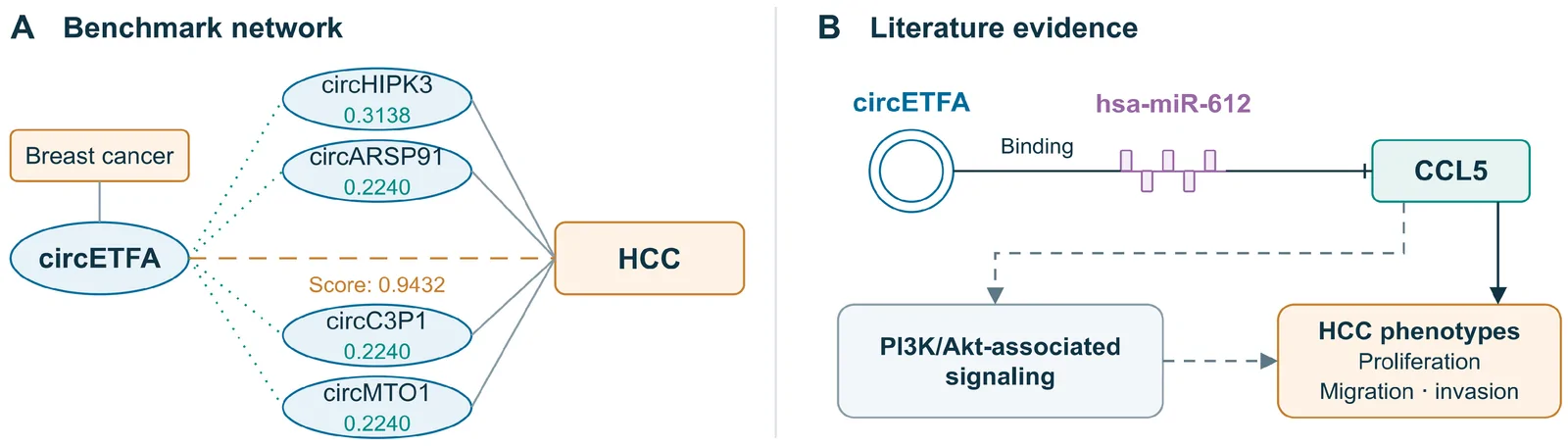
Network context and literature-supported molecular relationships for circETFA in hepatocellular carcinoma (HCC). (**A**) Benchmark associations (solid gray lines), circRNA similarities (dotted teal lines), and the predicted circETFA–HCC association (dashed orange line; score, 0.9432). Values beside neighboring circRNAs denote similarity to circETFA. (**B**) Literature-supported circETFA–miR-612–CCL5 relationships and PI3K/Akt-associated signaling in HCC [40]. The inhibitory bar denotes miRNA-mediated repression; dashed gray arrows indicate pathway context rather than a direct regulatory relationship established by MHDA.

To provide a disease-specific illustration, Figure 7A shows the benchmark neighborhood of the circETFA–hepatocellular carcinoma (HCC) candidate, with a prediction score of 0.9432. In the benchmark, circETFA is connected to breast cancer and shares similarity with HCC-associated circRNAs, including circHIPK3, circARSP91, circC3P1, and circMTO1. The displayed similarity values describe relationships in the benchmark-derived representation; they are not feature-attribution scores.

Figure 7B summarizes the published molecular context of circETFA in HCC. Lu et al. used RNA pull-down and luciferase reporter assays to support circETFA binding to miR-612 and miR-612 targeting CCL5 [40]. Their findings suggest that circETFA relieves miR-612-mediated repression of CCL5. CCL5 knockdown partly reduced the increases in HCC cell proliferation and migration associated with circETFA overexpression. Changes in Akt phosphorylation and downstream proteins also supported a connection with PI3K/Akt signaling. These published molecular relationships and pathway findings provide biological context for interpreting the circETFA–HCC candidate; they are not separate molecular predictions or experimental findings generated by MHDA.

## 4. Discussion

The results suggest that integrating complementary similarity views and heterogeneous relationships can improve the prioritization of potential CDAs. The ablation analyses support the contributions of the main MHDA components, while the case study provides literature-based context for selected predictions. Such agreement supports biological plausibility but does not establish the biological role of a predicted association.

Differences in negative sampling, class balance, and evaluation procedures can affect performance metrics, particularly AUPR and F1. These factors should be considered when interpreting the results summarized in Table 3.

The multi-view encoder combines information from distinct similarity graphs, and the heterogeneous module connects within-type and cross-type relationships. The dual-stream decoder retains both element-wise interactions and joint pair context. These design choices offer complementary ways to represent candidate associations. However, the model scores and graph neighborhoods should be interpreted as computational evidence for prioritization, rather than direct explanations of molecular regulation.

The current cross-validation evaluates unknown associations within fixed sets of circRNA and disease nodes. It does not directly assess cold-start prediction for previously unseen nodes, for which association-derived similarities may provide limited information. Cold-start evaluation and additional association-independent features are therefore important directions for future work. Alternative negative-sampling strategies may also help assess the dependence of the reported performance on the sampled unobserved pairs.

Generalization beyond the benchmark remains to be established. The current model does not directly incorporate miRNA, lncRNA, protein interaction, or pathway data as molecular features. The literature-based case analysis also requires follow-up biological experiments. Independent datasets and patient cohorts would be needed to assess external validity and potential biomarker applications, as illustrated by recent work on circulating circRNA panels for pancreatic cancer detection [41]. Integrating additional biological priors and evaluating them in independent settings are priorities for future work.

## 5. Conclusions

MHDA combines multi-view graph attention, heterogeneous relation enhancement, dual-stream decoding, and contrastive regularization for circRNA–disease association prediction. The reported benchmark performance and literature-supported case analysis suggest that the framework can help prioritize candidates for further investigation. Its broader utility will require evaluation on independent data and experimental study of selected predictions.

## Figures and Tables

**Table 1:** Summary statistics of the collected CDA datasets.

Database	CircRNAs	Diseases	Associations
CircR2Disease	561	100	607
CircAtlas	798	117	775
Circ2Disease	234	60	254
CircRNADisease	286	48	304

**Table 2:** Five-fold Cross-validation Performance.

Fold	AUC	AUPR	F1	Precision	Recall	ACC
1	0.9691	0.9662	0.9333	0.8947	0.9754	0.9303
2	0.9627	0.9534	0.9280	0.9062	0.9508	0.9262
3	0.9622	0.9571	0.9195	0.8571	0.9917	0.9132
4	0.9607	0.9627	0.9098	0.9024	0.9173	0.9090
5	0.9619	0.9602	0.8988	0.8219	0.9917	0.8884
Mean	0.9633	0.9599	0.9179	0.8764	0.9654	0.9134
SD	0.0029	0.0043	0.0124	0.0323	0.0283	0.0147

**Table 3:** Comparison of MHDA with published results for CDA prediction on CircR2Disease.

Category	Model	Brief Description	AUC	AUPR	F1
Graph reasoning and matrix completion methods	TCGIBMT	Integrates multi-source similarities and performs prediction by combining dual-channel graph reasoning with matrix completion.	0.9357	0.0838	0.1933
	Bi-SGTAR	Constructs a dual-view model based on the association matrix and exploits sparse gating and supervised reconstruction to uncover latent associations.	0.8747	0.0100	0.0107
Heterogeneous graph representation learning methods	HGECDA	Constructs a circRNA–miRNA–disease heterogeneous network and learns node representations through graph embedding.	0.9032	0.9123	0.8523
	HGCNCDA	Uses high-order graph convolution to integrate multiple biological relationships and capture long-range topological dependencies.	0.9491	0.5302	0.5532
	GATCL2CD	Combines heterogeneous graph attention with feature convolution to enhance high-order representation capability.	0.9355	0.0756	0.0180
Multi-source/multi-view feature fusion methods	RPMVCDA	Integrates multi-view graph convolution, stochastic perturbation networks, and a self-attention mechanism for prediction.	0.9518	0.9569	0.9110
	MNDCDA	Combines multi-source domain knowledge embedding with feature projection networks to learn association representations.	0.9070	0.2376	0.0187
Ours	MHDA	Integrates multi-source information and performs prediction via multi-view encoding, heterogeneous enhancement, and dual-stream decoding.	0.9633	0.9599	0.9179

Results for the seven comparison methods are taken from their original publications; MHDA results are from this study.

**Table 4:** Literature support for the top 25 ranked CDA candidates.

Rank	CircRNA	Disease	PMID/Status
1	hsa_circ_0001727	Hepatocellular carcinoma	36691031
2	hsa_circ_001763	Hepatocellular carcinoma	35400275
3	hsa_circ_0000175	Systemic lupus erythematosus	32093518
4	circ-FBXW7	Lung adenocarcinoma	37393311
5	hsa_circ_0003838	Breast cancer	unconfirmed
6	hsa_circ_0000518	Breast cancer	35112991
7	hsa_circ_0001946	Esophageal cancer	30674324
8	hsa_circ_0008717	Hepatocellular carcinoma	32196586
9	hsa_circ_0067934	Gestational diabetes mellitus	unconfirmed
10	hsa_circ_0008433	Intracranial aneurysms	31254341
11	Circ SLC8A1	Urinary bladder cancer	31228937
12	hsa circ 0006404	Endometrial cancer	36628205
13	circRHOBTB3	Gastric cancer	31928527
14	Circ_HIPK3	Glioblastoma	34198978
15	circHIAT1	Hepatocellular carcinoma	31108351
16	circPVT1	Acute myeloid leukemia	36718632
17	circ_RIMS1	Urinary bladder cancer	33230478
18	hsa_circ_0002024	Urinary bladder cancer	30972190
19	hsa_circ_0001946	Lung cancer	30841451
20	hsa_circ_0001946	Osteosarcoma	30425578
21	hsa_circ_0000219	Colorectal cancer	unconfirmed
22	hsa_circ_0023404	Endometrial cancer	36352482
23	circBCL11B	Oral squamous cell carcinoma	34288804
24	hsa_circ_0000175	Rheumatoid arthritis	32124964
25	circETFA	Hepatocellular carcinoma	34716323

## Data Availability

The data sets and source code can be freely downloaded from https://github.com/yhzhou165-netizen/MHDA.git (accessed on 1 November 2026).
